# Efficacy of Thymosin Alpha 1 in the Treatment of COVID-19: A Multicenter Cohort Study

**DOI:** 10.3389/fimmu.2021.673693

**Published:** 2021-08-02

**Authors:** Jiao Liu, Yanfei Shen, Zhenliang Wen, Qianghong Xu, Zhixiong Wu, Huibin Feng, Zhongyi Li, Xuan Dong, Sisi Huang, Jun Guo, Lidi Zhang, Yizhu Chen, Wenzhe Li, Wei Zhu, Hangxiang Du, Yongan Liu, Tao Wang, Limin Chen, Jean-Louis Teboul, Djillali Annane, Dechang Chen

**Affiliations:** ^1^Department of Critical Care Medicine, Ruijin Hospital, Shanghai Jiao Tong University School of Medicine, Shanghai, China; ^2^Department of Critical Care Medicine, Zhejiang Hospital, Hangzhou, China; ^3^Department of Surgical Intensive Care Unit, Huadong Hospital Affiliated to Fudan University, Shanghai, China; ^4^Intensive Care Unit, Huangshi Central Hospital, Affiliated Hospital of Hubei Polytechnic University, Huangshi, China; ^5^Department of Critical Care Medicine, Wuhan No.9 Hospital, Wuhan, China; ^6^Tuberculosis and Respiratory Department, Wuhan Jinyintan Hospital, Wuhan, China; ^7^Intensive Care Unit, Huazhong University of Science and Technology Union Jiangbei Hospital, Wuhan, China; ^8^Intensive Care Unit, Tianyou Hospital Affiliated to Wuhan University of Science & Technology, Wuhan, China; ^9^Service de Médecine-Intensive Réanimation, Hôpital Bicêtre, AP-HP. Université Paris-Saclay, Inserm UMR 999, Université Paris-Saclay, Le Kremlin-Bicêtre, France; ^10^Department of Intensive Care, Hôpital Raymond Poincaré (APHP), Laboratory of Infection & Inflammation – U1173, School of Medicine Simone Veil, University Versailles Saint Quentin – University Paris Saclay, INSERM, Garches, France

**Keywords:** thymosin alpha 1, COVID-19, non-recovery, disease severity, efficacy

## Abstract

**Background:**

Thymosin alpha 1 (Tα1) is widely used to treat patients with COVID-19 in China; however, its efficacy remains unclear. This study aimed to explore the efficacy of Tα1 as a COVID-19 therapy.

**Methods:**

We performed a multicenter cohort study in five tertiary hospitals in the Hubei province of China between December 2019 and March 2020. The patient non-recovery rate was used as the primary outcome.

**Results:**

All crude outcomes, including non-recovery rate (65/306 *vs.* 290/1,976, *p* = 0.003), in-hospital mortality rate (62/306 *vs.* 271/1,976, *p* = 0.003), intubation rate (31/306 *vs.* 106/1,976, *p* = 0.001), acute respiratory distress syndrome (ARDS) incidence (104/306 *vs.* 499/1,976, *p* = 0.001), acute kidney injury (AKI) incidence (26/306 *vs.* 66/1,976, *p* < 0.001), and length of intensive care unit (ICU) stay (14.9 ± 12.7 *vs.* 8.7 ± 8.2 days, *p* < 0.001), were significantly higher in the Tα1 treatment group. After adjusting for confounding factors, Tα1 use was found to be significantly associated with a higher non-recovery rate than non-Tα1 use (OR 1.5, 95% CI 1.1–2.1, *p* = 0.028). An increased risk of non-recovery rate associated with Tα1 use was observed in the patient subgroups with maximum sequential organ failure assessment (SOFA) scores ≥2 (OR 2.0, 95%CI 1.4–2.9, *p* = 0.024), a record of ICU admission (OR 5.4, 95%CI 2.1–14.0, *p* < 0.001), and lower PaO2/FiO2 values (OR 1.9, 95%CI 1.1–3.4, *p* = 0.046). Furthermore, later initiation of Tα1 use was associated with a higher non-recovery rate.

**Conclusion:**

Tα1 use in COVID-19 patients was associated with an increased non-recovery rate, especially in those with greater disease severity.

## Introduction

Coronavirus 2019 disease (COVID-19) represents an ongoing global threat to human health and has caused more than 2,400,000 deaths to date (JHU data-23/02/2021, https://coronavirus.jhu.edu/). As the COVID-19 outbreak continues, novel and effective therapies are urgently needed.

Current evidence indicates that immune dysfunction in COVID-19 plays a critical role in disease severity and is associated with a poor prognosis. Several studies have reported that inflammatory markers such as C-reactive protein (CRP), ferritin, interleukin (IL)-6, IL-10, and tumor necrosis factor-α (TNF-α) were significantly elevated in severe COVID-19 cases. The resulting cytokine storm is thought to cause rapid COVID-19 progression and increased deaths (1–3). Furthermore, Yu and colleagues (4) concluded that adaptive immune response dysregulation contributes to the cytokine storm and further leads to severe COVID-19. Dozens of studies have been performed to explore potential medical interventions to attenuate the harmful immune response, including statins (5), corticosteroids (6, 7), and thymosin alpha 1 (Tα1) (8–11). However, the benefits of immunomodulatory treatment of patients with COVID-19 have remained controversial.

Tα1 is an endogenous polypeptide hormone secreted by thymic epithelial cells (12). As an immunomodulatory therapy (12, 13), Tα1 has been investigated in many diseases involving immune dysfunction [such as sepsis, cystic fibrosis, and hepatitis viral infection (14–16)] and is associated with an improved patient outcome. The efficacy of Tα1 in COVID-19 has also been investigated in several studies (8–11). However, the conclusions remain inconsistent. For instance, in a retrospective study including 76 severe COVID-19 cases (8), Liu et al. reported that Tα1 use could increase T cell numbers and was associated with reduced mortality. However, other studies found that Tα1 use showed no effect on mortality in COVID-19 (9) or was even associated with increased mortality in patients with COVID-19 of all severity levels (10). The reasons behind these inconsistent findings remain unclear. However, emerging evidence indicated that COVID-19 is a heterogeneous disease (17, 18). Whether the role of Tα1 in COVID-19 is affected by different phenotypes remains unclear.

To this end, we performed a large multicenter cohort study to explore the efficacy of Tα1 in COVID-19 patients displaying a range of disease severities. Interaction analysis between Tα1 and predefined parameters was also performed to identify the heterogeneous effectiveness of Tα1 in different patient subgroups.

## Methods

### Study Setting and Design

This was a multicenter cohort study conducted at five tertiary hospitals (in the Hubei province of China) that were designated by the Chinese government as COVID-19 treatment sites: including Union Jiangbei Hospital, Wuhan No. 9 Hospital, Wuhan No. 4 Hospital, Wuhan Jinyintan Hospital, and Huangshi Central Hospital. This study was approved by the Ethics Committee of the Jin Yin-tan Hospital (KY-2020-03.01), and patient informed consent was waived due to the retrospective nature of this study. All the patients admitted to the five tertiary hospitals between December 29, 2019 and March 16, 2020 were screened and included in the study if the following inclusion criteria were met: 1. Confirmed COVID-19 diagnosis made according to the Chinese COVID-19 diagnosis and treatment guidelines (Trial Version 7) (19); and 2. Age over 18 years. Patients were excluded if their hospital record was incomplete. No other exclusion criteria were applied.

### Data Extraction

Demographic characteristics, smoking and comorbidities data, and initial laboratory indices recorded within the first 24 h after hospital admission were extracted from each center through electronic medical records. The change in lymphocyte counts was defined as the difference between the initial lymphocyte count and the minimum lymphocyte count. Clinical classification, special drug combinations, and clinical outcomes data were also recorded. The sequential organ failure assessment (SOFA) scores (20) and acute physiology and chronic health evaluation II (APACHE II) scores (21) were calculated to assess the severity of illness using data obtained in the first 24 h after hospital admission. Tα1 use was defined as any record of subcutaneous/intramuscular Tα1 administration throughout the entire hospital stay. Both the duration and timing of Tα1 use were also extracted.

### COVID-19 Severity Classification

The detailed diagnosis of COVID-19 (19) was established as follows: 1. patients with an epidemiological history and chest imaging (computed tomography or radiography) that suggested viral pneumonia; 2. laboratory-confirmed SARS-COV-2 infection of throat swab, sputum, and/or lower respiratory tract samples by high-throughput sequencing or real-time reverse transcriptase polymerase chain reaction (RT-PCR); or 3. confirmed plasma positivity for specific antibody (IgM or/and IgG) against SARS-COV-2.

COVID-19 disease severity was classified according to the Chinese diagnosis and COVID-19 treatment guidelines (Trial Version 7) (19). Briefly, patients were diagnosed with moderately severe COVID-19 if they had fever, respiratory symptoms, and chest imaging (computed tomography or radiography) suggesting viral pneumonia. Patients who met either conditions i–iii or iv–vi of the following criteria were diagnosed as having severe or critical disease, respectively: (i) respiratory rate ≥30 beats/min; (ii) resting stable oxygen saturation (SpO2) ≤93%; (iii) PaO2/FiO2 ≤300 mmHg (1 mmHg = 0.133 kPa); (iv) respiratory failure requiring mechanical ventilation; (v) shock; or (vi) multiple organ failure requiring ICU admission.

Diagnosis of acute respiratory distress syndrome (ARDS) was made according to the Berlin definition (22). Acute kidney injury (AKI) was defined according to the “Kidney Disease: Improving Global Outcomes” document (23).

### Stratification and Outcome Definition

Subgroup analysis was performed to interpret possible interactions between Tα1 use and disease severity. Data were stratified according to the median of the maximum SOFA score (≥/<2), PaO2/FiO2 at admission, admission to intensive care unit (ICU), application of mechanical ventilation, and development of ARDS. Non-recovery rate was the primary outcome at the time of data collection. Non-recovering patients were those who died as a result of COVID-19 or who were still in hospital, but in any one of severely deteriorated condition: a. after sufficient fluid resuscitation (≥30 ml/kg crystalloid solution), a dose of vasopressor therapy equal to or greater than an equivalent of norepinephrine 0.4 μg/kg/min for at least 24 h was hard to maintain mean arterial pressure (MAP) at 65 mmHg; b. PaO2/FiO2 was continuously lower than 100 mmHg for 24 h even with invasive mechanical ventilation. Secondary outcomes included in-hospital mortality, ICU admission, intubation rate, ARDS incidence, AKI incidence, duration of mechanical ventilation, length of ICU stay, and length of hospital stay. Sensitivity analysis was also performed using in-hospital mortality as the primary outcome.

### Management of Missing Data

Most continuous variables in the study had less than 5% missing data and were therefore replaced with their mean or median values. Variables with more than 20% missing data were not completed. For dichotomous variables, the missing value was replaced with the default value (zero).

### Statistical Analysis

Continuous variables were presented as mean ± standard deviation (SD) or median (interquartile range, IQR) according to the data distribution. The Student’s t-test or Wilcoxon rank-sum test were used as appropriate. Categorical data were compared using the Chi-squared test and were presented as percentages. To adjust for potential confounding factors, variables with *p* < 0.2 in the univariate comparison were included in the initial model. Stepwise regression was used to build the final logistic model. Multicollinearity was tested using the variance inflation factor (VIF) method, with a VIF ≥ 5 suggesting potential multicollinearity. To test the stability of these logistic models, bootstrap analysis (using 100 resamples) was performed.

The significant impact of maximum SOFA scores on the association between Tα1 use and the non-recovery rate was detected in the multivariable analysis. Thus, interactions between Tα1 use and disease severity indices were explored, and subgroup analysis was performed according to the following five parameters: median SOFA score (≥/<2), ICU admission, PaO2/FiO2 value, application of mechanical ventilation, and development of ARDS. For interpretation, the predicted marginal effect of Tα1 was estimated at different SOFA scores and PaO2/FiO2 values.

Propensity score matching (PSM) was applied to minimize the impact of confounding factors, such as biochemical indices and the disease severity score, which may lead to a biased outcome. The propensity score was assigned based on the probability that a patient would receive Tα1 therapy and estimated using a multivariable logistic regression model. A one-to-three nearest neighbor matching algorithm was applied with a caliper of 0.02. The following variables were selected to generate the propensity score: age, hypertension, chronic obstructive pulmonary disease (COPD), chronic liver disease, chronic cardiac disease, maximum SOFA score, ICU admission, serum hemoglobin and creatinine levels, and initial white blood cell, lymphocyte, and platelet counts. The bias in the means (or proportions) of covariates between two groups was examined using the standardized difference before and after PSM. Finally, to test bias owing to imbalances in unmeasured covariates in the PSM, sensitivity analyses were performed to quantify the degree of hidden bias (Gamma value) that would need to be present to invalidate our main conclusions. A two-tailed test was performed, and *p* < 0.05 was considered statistically significant. All statistical analyses were performed using STATA 14.0 (College Station, TX, USA).

## Results

### Baseline Characteristics and Crude Comparisons

**Figure 1:**
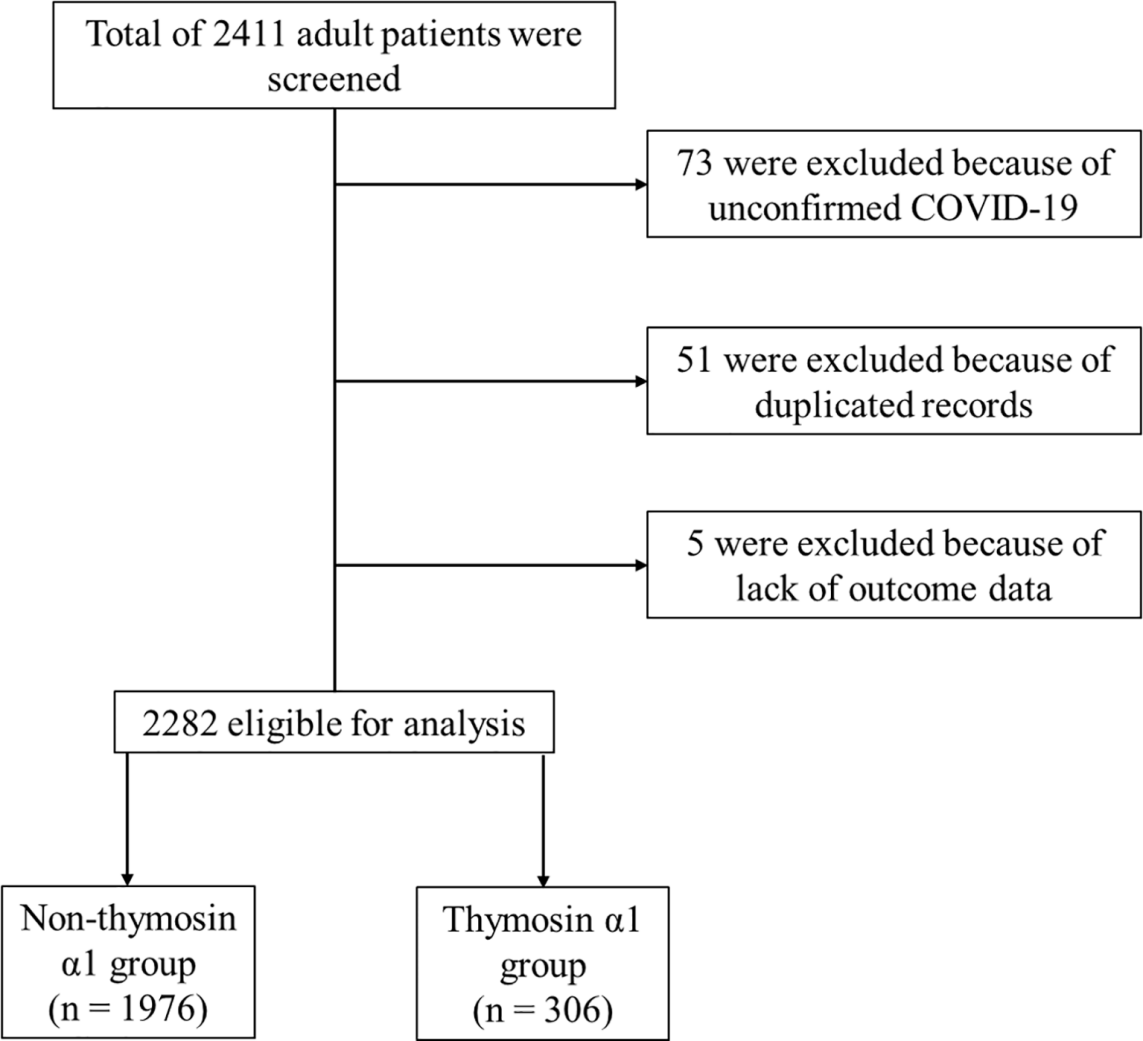
Flow chart of the present study.

Of the 2,411 patients admitted to the five participating tertiary hospitals, 73 were excluded because of unconfirmed COVID-19, 51 were excluded because of duplicate records, and 5 were excluded due to missing outcome data. Of the remaining 2,282 patients that were eligible to participate in our study, 306 received Tα1 therapy (**Figure 1**). Clinical characteristics associated with COVID-19 patients in the Tα1 and non-Tα1 treatment groups are shown in **Table 1**. There were no significant differences in terms of age or gender between the two groups. More patients received corticosteroids (107/306 *vs.* 382/1,976, *p*<0.001) and interferon (74/306 *vs.* 108/1,976, *p*<0.001) therapy in the Tα1 group than in the non-Tα1 group. Patients who received Tα1 therapy had almost the same disease severity according to the SOFA score, APACHE II score, initial serum creatinine and maximum lactate levels, although the patients in the Tα1 group included a slightly larger proportion of critically ill patients (59/306 *vs.* 240/1,976). Furthermore, although both the initial and minimal lymphocyte counts were significantly lower in the Tα1 group than in the non-Tα1 group (1.0 ± 0.5 *vs.* 1.1 ± 0.6 × 10^9/L, *p* = 0.007; 0.8 ± 0.5 *vs.* 0.9 ± 0.6 × 10^9/L, *p* < 0.001 for the Tα1 and non-Tα1 groups, respectively), the change in lymphocyte count was comparable between the groups (0.1 ± 0.3 *vs.* 0.1 ± 0.3 × 10^9/L, *p* = 0.337). However, as reported in **Table 2**, all crude outcomes, including non-recovery rate (65/306 *vs.* 290/1,976, *p* = 0.003), in-hospital mortality rate (62/306 *vs.* 271/1,976, *p* = 0.003), intubation rate (31/306 *vs.* 106/1,976, *p* = 0.001), ARDS incidence (104/306 *vs.* 499/1,976, *p* = 0.001), AKI incidence (26/306 *vs.* 66/1,976, *p* < 0.001), and Length of ICU stay (14.9 ± 12.7 *vs.* 8.7 ± 8.2 days, *p* < 0.001), were significantly higher in the Tα1 group than in the non-Tα1 group. Information relating to the number of patients, Tα1 use, and the non-recovery rate for each center is provided in **Supplementary Table S1**.

**Table 1:** Baseline comparisons between thymosin α1 and non-thymosin α1 groups.

Variables	Non-thymosin α1 group (n = 1,976)	Thymosin α1 group (n = 306)	*p*
**Age (years)**	58.4 ± 14.5	57.9 ± 14.5	0.601
**Gender, n (%)**			
Male	1025 (51.9)	165 (53.9)	0.504
Female	951 (48.1)	141(46.1)	
**Smoking, n (%)**	54 (2.7)	7 (2.2)	<0.001
**Comorbidities, n (%)**			
Hypertension	525 (26.5)	98 (32.0)	0.046
Diabetes mellitus	247 (12.5)	36 (11.7)	0.717
Chronic heart diseases	182 (9.2)	18 (5.8)	0.055
Chronic obstructive pulmonary disease	39 (1.9)	9 (2.9)	0.272
Chronic renal diseases	54 (2.7)	6 (1.9)	0.432
Malignant tumor	50 (2.5)	7 (2.2)	0.800
**Disease severity scores [median (IQR)]**			
SOFA score on admission	1 (0–2)	1 (0–2)	0.915
Maximum SOFA score	1 (0–2)	1 (0–3)	0.926
APACHE II on admission	5 (3–7)	5 (3–7)	0.949
Maximum APACHE II score	5 (3–7)	5 (3–8)	<0.001
**Clinical classification, n (%)**			<0.001
Moderate type	1276 (64.6)	186 (60.8)	
Severe type	460 (23.3)	61 (19.9)	
Critical type	240 (12.1)	59 (19.3)	
**Laboratory finding**			
PaO2/FiO2	279.3 ± 144.2	316.1 ± 107.7	0.002
Initial white blood cell count (10^9/L)	6.9 ± 3.6	6.9 ± 4.0	0.977
Initial lymphocyte cell count (10^9/L)	1.1 ± 0.6	1.0 ± 0.5	0.007
Minimum lymphocyte cell count (10^9/L)	0.9 ± 0.6	0.8 ± 0.5	<0.001
Change of lymphocyte cell count (10^9/L)	0.1 ± 0.3	0.1 ± 0.3	0.337
Initial hemoglobin level (g/dl)	122.7 ± 17.0	123.4 ± 18.2	0.462
Initial platelet count (10^9/L)	217.4 ± 89.5	207.5 ± 87.0	0.071
Initial albumin level (g/l)	34.3 ± 5.0	32.4 ± 4.8	<0.001
Initial serum creatinine (mmol/l)	75.9 ± 51.9	82.1 ± 66.6	0.063
Initial serum sodium (mmol/L)	139.7 ± 4.2 (n = 1,606)	140.8 ± 3.2 (n = 261)	<0.001
Maximum lactate level (mmol/L)	2.5 ± 2.4 (n = 366)	2.4 ± 2.0 (n = 10)	0.913
**Medicine, n (%)**			
Corticosteroids	382 (19.3)	107 (35.0)	<0.001
Interferon	158 (8.0)	74 (24.2)	<0.001
Ribavirin	95 (4.8)	4 (1.3)	0.005
Oseltamivir	46 (2.3)	6 (2.0)	0.689

PaO2/FiO2, ratio of partial pressure of arterial oxygen to fraction of inspired oxygen; SOFA, sequential organ failure assessment; APACHE II, acute physiology and chronic health evaluation II.

**Table 2:** Clinical outcomes of overall COVID-19 patients with or without thymosin α1 treatment.

Clinical outcomes	Non-thymosin α1 group (n = 1,976)	Thymosin α1 group (n = 306)	*p*
**Primary outcome**			
Non-recovery, n (%)	290 (14.6)	65 (21.2)	0.003
**Secondary outcomes**			
In-hospital mortality, n (%)	271 (13.7)	62 (20.2)	0.003
ICU admission, n (%)	287 (14.5)	42 (13.7)	0.711
Intubation, n (%)	106 (5.3)	31 (10.1)	0.001
Acute respiratory distress syndrome, n (%)	499 (25.2)	104 (33.9)	<0.001
Acute kidney injury, n (%)	66 (3.3)	26 (8.4)	<0.001
Duration of mechanical ventilation (days)	6.2 ± 4.8	6.7 ± 4.2	0.644
Length of ICU stay (days)	8.7 ± 8.2	14.9 ± 12.7	<0.001
Length of hospital stay (days)	13.8 ± 7.9	12.1 ± 8.2	<0.001

ICU, intensive care unit.

### Adjusted Association Between Tα1 Use and Non-Recovery Rate

Potential confounding factors were adjusted for in the multivariable logistic models as shown in the **Table 3**. The odds ratio (OR) for the non-recovery rate of Tα1 use was found to be significant (Model 2: OR 1.5, 95% confidence interval (CI) 1.1–2.1, *p* = 0.028) using bootstrap analysis with 1,000 resamples. However, after the addition of the maximum SOFA score to Model 3, the association between Tα1 use and the non-recovery rate became non-significant (OR 1.4, 95% CI 0.9–2.2, *p* = 0.118).

**Table 3:** Associations between non-recovery rate and thymosin α1 use in different logistic models.

Variables	Model 1		Model 2		Model 3	
	Crude OR (95% CI)	*p*	Multivariable logistic model with bootstrapping, aORs (95% CI)	*p*	Adjusted aORs (95% CI)	*p*
Thymosin α1 use	1.5 (1.1 – 2.1)	0.003	1.5 (1.1–2.1)	0.028	1.4 (0.9–2.2)	0.118
Age >65			3.4 (2.6–4.4)	<0.001	3.9 (2.8–5.3)	<0.001
Diabetes mellitus			1.6 (1.1–2.2)	0.007	1.3 (0.8–2.0)	0.204
PaO2/FiO2 <300			2.3 (1.7–3.1)	<0.001	0.6 (0.4–0.9)	0.042
Lymphocyte counts			0.4 (0.3–0.5)	<0.001	0.5 (0.4–0.7)	<0.001
Platelet counts			0.9 (0.9–0.9)	<0.001	0.9 (0.9–0.9)	0.287
Creatinine level			1.0 (1.0–1.0)	0.001	0.9 (0.9–1.0)	0.108
Union Jiangbei Hospital			Ref.	-	Ref.	-
Wuhan No.9 Hospital			1.0 (0.6–1.6)	0.979	0.1 (0.06–0.2)	<0.001
Wuhan No.4 Hospital			3.0 (1.5–6.0)	0.002	0.5 (0.2–1.0)	0.062
Wuhan Jinyintan Hospital			1.4 (0.9–2.3)	0.117	0.1 (0.1–0.3)	<0.001
Huangshi Central Hospital			6.0 (2.6–14.2)	<0.001	0.4 (0.2–1.2)	0.114
Maximum SOFA score					1.9 (1.8–2.1)	<0.001

Three logistic models were used to evaluate the association between non-recovery rate and thymosin α1 use. OR of thymosin α1 use was significantly associated with increased non-recovery rate in models 1 and 2. However, after adjusting for SOFA score in model 3, the OR of thymosin α1 became non-significant. Bootstrap techniques (100 resamples) was used for calculating 95% CI in models 2 and 3.

OR, odds ratio; aORs, adjusted odds ratios; PaO2/FiO2, ratio of partial pressure of arterial oxygen to fraction of inspired oxygen; SOFA, sequential organ failure assessment.

**Figure 2:**
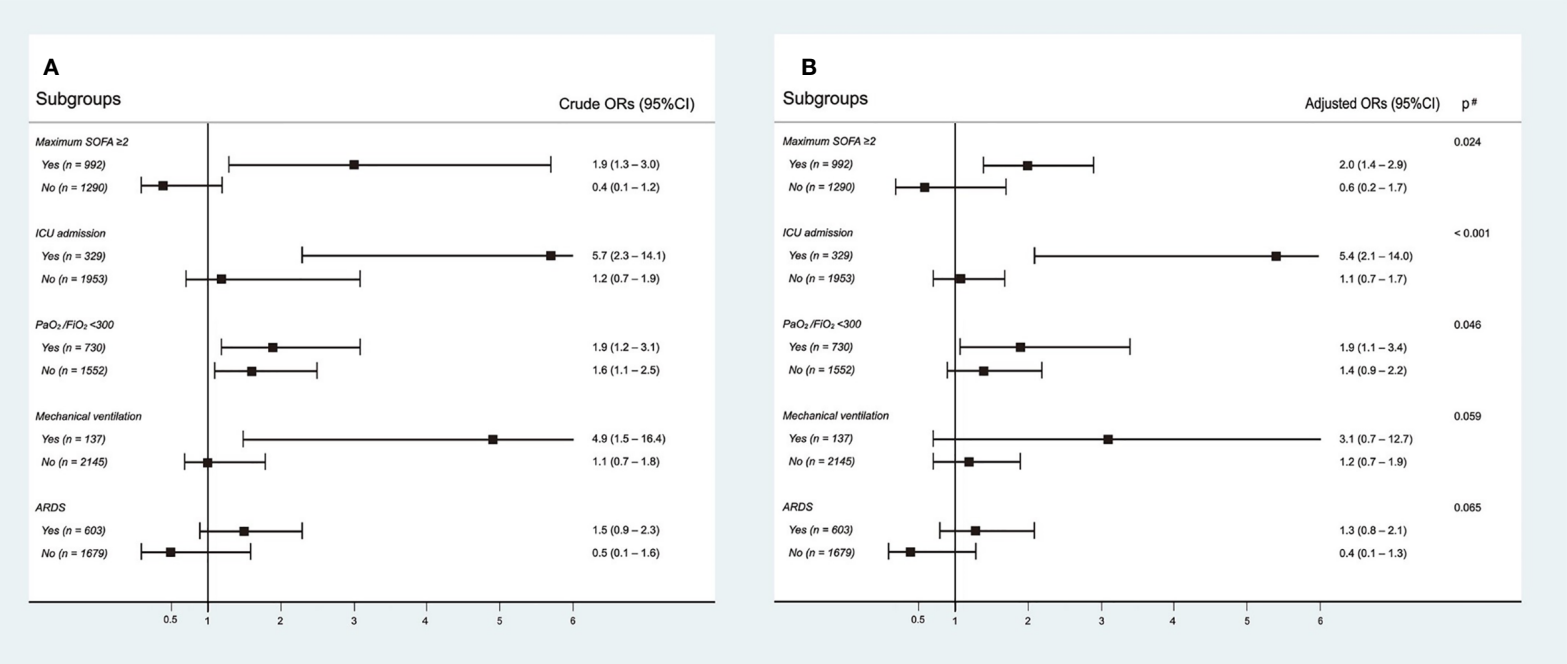
Interactions between thymosin-α1 use and different disease severity indexes. Subgroup analysis according to maximum SOFA score, ICU admission, PaO_2_/FiO_2_, mechanical ventilation, and development of ARDS. The crude outcomes are presented in **(A)**, and the adjusted outcomes are presented in **(B)**.

Interactions between Tα1 use and different disease severity indices were evaluated. There were significant interactions between Tα1 use and the maximum SOFA score (*p* = 0.024) and ICU admission (*p* < 0.001) (**Figure 2**). Tα1 use was significantly associated with an increase in non-recovery rate in the subgroup with a maximum SOFA score ≥2 (OR 2.0, 95% CI 1.4–2.9) or ICU admission (OR 5.4, 95% CI 2.1–14.0), whereas this association was not significant in subgroups with a SOFA score <2 (OR 0.6, 95% CI 0.24–1.7) or without ICU admission (OR 1.1, 95% CI 0.7–1.7). There was no significant interaction between Tα1 use and PaO2/FiO2 values (≥300 mmHg) at admission, mechanical ventilation, or ARDS development. However, a trend towards higher ORs was observed in subgroups with low PaO2/FiO2 values (<300 mmHg), mechanical ventilation, and ARDS. Furthermore, significant interactions were also observed using maximum SOFA (*p* = 0.003) and PaO2/FiO2 values at admission (*p* = 0.016) as continuous variables. The predicted marginal effect of Tα1 on the non-recovery rate was estimated using different SOFA and PaO2/FiO2 values (**Supplementary Figure S1**). The difference in the probability of non-recovery between the Tα1 and non-Tα1 groups correlated positively with the SOFA score or negatively with the PaO2/FiO2 value. In addition, aiming to evaluate whether the effect of Tα1 administration was influenced by other concurrent immunotherapies, we tested the potential interaction between Tα1, corticosteroids, and interferon and found no significant interaction. We also included both the corticosteroids and interferon factors in our multivariable model, and the overall results in each subgroup remained unchanged (**Supplementary Table S2**). The proportions of patients receiving Tα1, corticosteroids, and/or interferon are presented in a Venn diagram (**Supplementary Figure S2**).

PSM was performed using a 1:3 algorithm; 306 cases from the Tα1 group and 720 cases from the non-Tα1 group were well matched (**Supplementary Tables S3**, **4**). The overall quality of the matched sample was assessed by comparing the standardized difference of included covariates before and after PSM. There was no significant difference between the matched groups relating to all 11 covariates (**Supplementary Table S3**). However, all clinical outcomes, including the non-recovery rate (65/306 *vs.* 111/720, *p* = 0.024), intubation rate (31/306 *vs.* 43/720, *p* = 0.018), ARDS incidence (104/306 *vs.* 196/720, *p* = 0.029), and AKI incidence (26/306 *vs.* 26/720, *p* = 0.001) were significantly higher in the Tα1 group than in the non-Tα1 group (**Table 4**). Furthermore, another PSM was performed in the subgroup with a maximum SOFA ≥ 2, as the impact of Tα1 use on the non-recovery rate was significantly affected by disease severity, and all the above conclusions were supported (**Table 4**).

**Table 4:** Comparisons of clinical outcomes after propensity score matching.

Clinical outcomes	All patients			Patients with SOFA ≥2		
	Non-thymosin α1 group(n = 720)	Thymosin α1 group(n = 306)	*p*	Non-thymosin α1 group(n = 302)	Thymosin α1 group(n = 142)	*p*
Non-recovery, n (%)	111 (15.4)	65 (21.2)	0.024	90 (29.8)	59 (41.5)	0.014
Intubation, n (%)	43 (5.9)	31 (10.1)	0.018	30 (9.9)	26 (18.3)	0.013
ARDS, n (%)	196 (27.2)	104 (33.9)	0.029	151 (50.0)	97 (68.3)	<0.001
AKI, n (%)	26 (3.6)	26 (8.5)	0.001	22 (7.2)	23 (16.2)	0.004

As heterogeneous effects of thymosin α1 was observed in patients with different disease severity levels,PSM was performed both in all patients and in patients with SOFA ≥ 2. The following variables were selected to generate the propensity score: age, ICU admission, COPD, hypertension, chronic cardiac disease, the initial white blood cell, lymphocyte,and platelet count, initial hemoglobin and creatinine level, and maximum SOFA score during hospital admission.

Gamma of sensitivity analysis was 1.2.

SOFA, sequential organ failure assessment.; ARDS, acute respiratory distress syndrome; AKI, acute kidney injury; CRRT, continuous renal replacement therapy.

### Sensitivity Analysis

In the non-recovery group (n = 355), there are 18 patients still under treatment, but in a severely deteriorated condition during data extraction. For robustness, we performed sensitivity analysis under three patterns, and “death” was used as the dependent outcome (**Supplementary Table S4**). In pattern 1, these 18 patients were divided into the “alive” group. In pattern 2, these 18 patients were divided into the “death” group. In pattern 3, these 18 patients were excluded from the analysis. The results were stable in all these three patterns. In addition, interactions between thymosin α1 use and disease severity indexes on in-hospital mortality are also presented in **Supplementary Table S5**.

### Association Between Duration/Timing of Tα1 Treatment and Patient Non-recovery Rate

Both the duration and timing of Tα1 were included in the multivariable logistic model in different forms (**Table 5**, Model 1: continuous variable; Model 2: dichotomous variable according to median value; and Model 3: tertile analysis). Tα1 use at a later stage was significantly associated with a higher non-recovery rate than Tα1 use at an earlier stage (Models 1–3). However, the duration of Tα1 use had a less significant effect on recovery rate. Inclusion of the maximum SOFA score as a confounding factor in the logistic model did not alter the results (**Supplementary Table S6** **)**.

**Table 5:** Association between the duration/timing of thymosin α1 use and non-recovery rate.

Duration of thymosin α1 use (days)	ORs	95% CI	*p*	Timing of thymosin α1 use (days)	ORs	95% CI	*p*
**Model 1**				**Model 1**			
Continuous variable (n = 306)	1.0	0.9–1.1	0.481	Continuous variable (n = 306)	1.1	1.0–1.2	<0.001
**Model 2** (median value)				**Model 2** (median value)			
≤5 (n = 172)	Ref.			≤3 (n = 159)	Ref.		
>5 (n = 134)	1.3	0.7–2.1	0.404	>3 (n = 147)	2.4	1.3–4.6	0.007
**Model 3** (tertile analysis) (*p* for trend: 0.574)				**Model 3** (tertile analysis) (*p* for trend: 0.005)			
≤3 (n = 106)	Ref.			≤1 (n = 109)	Ref.		
3–7 (n = 103)	0.4	0.2–0.9	0.037	2–10 (n = 91)	4.1	1.5–10.7	0.004
≥7 (n = 97)	1.2	0.6–2.5	0.648	≥10 (n = 106)	6.2	2.5–15.3	<0.001

All models were adjusted for age, diabetes, PaO2/FiO2, lymphocyte and platelet count, serum creatinine level.

ORs, odds ratios.

## Discussion

The current study has three major findings. First, Tα1 use in COVID-19 was associated with poor clinical outcomes. This finding was confirmed both in the multivariable logistic model and *via* PSM. Second, there were significant interactions between Tα1 use and disease severity indices, and the association between Tα1 use and the non-recovery rate was stronger in severe COVID-19 cases. Third, Tα1 use at a later stage was significantly associated with a higher non-recovery rate than Tα1 use at an earlier stage. However, the duration of Tα1 use had a less significant effect on recovery rate.

Lymphopenia has been commonly reported in patients with severe COVID-19 (4, 24, 25) and is associated with a poor outcome (24). Yu et al. (4) reported that a significant reduction in effector T cell numbers, accompanied by an increase in the frequency of effector B cells, was observed in COVID-19 patients with severe disease. This indicates that a decline in T cell numbers is likely the main cause of lymphopenia and may aggravate COVID-19 severity. Given the essential role of T cells in viral eradication, exploring medical interventions designed to boost T lymphocyte number and function may improve COVID-19 prognosis (26). Previous studies have indicated that Tα1 can promote T cell development and proliferation, increase their number, and enhance their function (27, 28). Yu’s research (4) also demonstrated that Tα1 promoted the proliferation of effector T cells *in vitro* and relieved lymphopenia in COVID-19 patients. Matteucci et al. (29) found that genes associated with cytokine signaling and expression were upregulated in patients with COVID-19, and the *ex vivo* treatment with Tα1-mitigated cytokine expression, and inhibited lymphocyte activation. These findings provide the basis for the rationale use of Tα1 in COVID-19, but these findings need to be further supported by clinical studies. A recent cohort study showed that Tα1 can reduce mortality in patients with severe COVID-19 by reversing lymphocytopenia and restoring the function of exhausted T cells (8). However, bias risk should be considered when interpreting this conclusion, due to the limited sample size used (only 76 patients with severe COVID-19 were included) and the presence of potential confounding factors. In another multicenter observation study, Wu et al. (11) also found that Tα1 significantly decreased 28-day mortality and attenuated acute lung injury in critical type COVID-19 patients. However, the definition of critical type is largely different from our study. In addition, the disease severity was significantly imbalanced between Tα1 and non-Tα1 groups, as patients in the Tα1 group had worse condition. Noteworthy, inconsistent findings were also reported in other studies. Sun et al. (9) reported that in 771 patients with COVID-19, use of Tα1 was not associated with decreased mortality in critically ill COVID-19 patients. Wang et al. conducted a retrospective propensity score matched study including 317 COVID-19 patients, treatments with immunomodulatory therapies, including glucocorticoids, immunoglobulin, and thymosin, were significantly associated with a higher rate of COVID-19 death (10).

In the current study, Tα1 was used in 13.4% of COVID-19 patients. We noted that Tα1 use was associated with poor prognoses, such as higher non-recovery and in-hospital mortality rate and higher ARDS and AKI incidence. After adjusting for potential confounders, Tα1 use was still significantly associated with an increased non-recovery rate. However, the underlying mechanism remains unclear. One study (30) reported that Tα1 exhibited a dual effect on dendritic cells (DCs) upon different pathogen encounter *in vitro*. Tα1 was shown to promote DCs to secrete inflammatory cytokines, such as TNF-α, IL-6, and IL-8, in response to viral infection. In contrast, Tα1 dampened inflammation when the DCs were exposed to bacterial pathogens. Furthermore, as a result of the interaction analysis, we found that Tα1 use was associated with increased non-recovery rate mainly in patient subgroups with greater disease severity. Recent studies have found that COVID-19 follows a heterogeneous course of disease (17, 18). Shi et al. (31) categorized COVID-19 cases into three stages based on inflammatory status: stage I, an asymptomatic incubation period; stage II, non-severe symptomatic period; and stage III, severe respiratory symptomatic stage. For patients in stage III, a hyper-inflammatory response characterized by the overexpression of inflammatory factors and cytokines played a key role in patient deterioration (32). During this third stage, immunosuppressive therapy as opposed to immune enhancement therapy is needed (31). However, studies have indicated that Tα1 plays an important role in the activation and maturation of DCs, which in turn increases the secretion of inflammatory factors, promotes the differentiation of T cell precursors into Th1, eventually exacerbating the inflammatory response to viral infection (28, 33). In our study, the greatest disease severity indices, such as higher SOFA scores, lower PaO2/FiO2 values, or admission to ICU, to a certain degree represented a more severe inflammatory stage. This may be the potential mechanism explaining why adjuvant Tα1 use was associated with poor outcomes especially in severe COVID-19 cases.

In addition, we found that the timing of Tα1 treatment is also crucial as applying Tα1 at a later stage was significantly associated with increased non-recovery rate. Aiming for robustness, the timing of Tα1 was included in the multivariable logistic model in three different forms, and the findings remain stable. We inferred that the inflammatory response may be stronger in the later stage than in the early stage in COVID-19. Therefore, compared to early use, Tα1 use at a later stage may exacerbate the inflammatory response and lead to a poor prognosis. However, data about the inflammatory status at different stages were lacking. More studies are needed to validate our speculation. In addition, we also investigated the impact of Tα1 duration on prognosis in COVID-19. In the tertile analysis, we noted that compared to Tertile 1 (≤3 days), OR in Tertile 2 was significant while non-significant in Tertile 3. When we included the duration of Tα1 in other two forms, the results became non-significant. Therefore, there is a chance that this significant finding may be affected by different cut-off values, and the stability of this result should be verified.

### Strengths and Limitations

To the best of our knowledge, this is the first large, multicenter observational study confirming the association between Tα1 therapy and increased non-recovery rate in severely ill COVID-19 patients. The main strengths of this study included the large sample size and the use of multiple statistical analyses methods to minimize confounding bias. However, several limitations need to be addressed. First, the use of Tα1 for the treatment of COVID-19 patients was mainly at the discretion of clinicians. Thus, the reasons for its use may have been quite varied, such as poor immune status, presence of hepatic disease, severe infection, or simply the clinician’s preference. Second, we included most of the covariates, both in a multivariable model and PSM analysis. However, bias caused by potential confounding factors remains possible, such as the time of COVID-19 diagnosis. Third, owing to the retrospective nature of this study, the protocol for Tα1 administration was diverse among patients; for instance, the timing of Tα1 use was significantly associated with a poor outcome, whereas the duration was not. Thus, rigorously designed randomized controlled studies would be required to reach a more precise conclusion. Fourth, the causal relationship between Tα1 and a poor clinical outcome in the context of COVID-19 cannot be inferred from the current study. Although the results from the PSM supported our hypothesis, our work is observational. Therefore, future prospective studies are needed to verify our results.

## Conclusions

The hyper-inflammatory response is a hallmark of COVID-19. Tα1 use in these patients is significantly associated with an increased non-recovery rate and a poor clinical outcome, especially in individuals with severe disease. As the COVID-19 outbreak is still ongoing, Tα1 use in this group of patients should be treated with caution.

## Data Availability Statement

The raw data supporting the conclusions of this article will be made available by the authors, without undue reservation.

## Ethics Statement

The studies involving human participants were reviewed and approved by the Ethics Committee of the Jin Yin-tan Hospital (KY-2020-03.01). Written informed consent for participation was not required for this study in accordance with the national legislation and the institutional requirements.

## Author Contributions

DC and XD designed the project. YC, LZ, JG, HD, YL,TW, LC, WL, and WZ carried out the data collection. YS analyzed the data and prepared the figures. JL, YS, ZLW, QX, ZXW, HF and ZL drafted the manuscript. JT and DA provided some writing suggestions. All authors contributed to the article and approved the submitted version.

## Funding

This work was supported by the National Natural Science Foundation of China [81873944, 81971869, and 81571943].

## Conflict of Interest

The authors declare that the research was conducted in the absence of any commercial or financial relationships that could be construed as a potential conflict of interest.

## Publisher’s Note

All claims expressed in this article are solely those of the authors and do not necessarily represent those of their affiliated organizations, or those of the publisher, the editors and the reviewers. Any product that may be evaluated in this article, or claim that may be made by its manufacturer, is not guaranteed or endorsed by the publisher.

## Abbreviations

SOFA, sequential organ failure assessment; ICU, intensive care unit; PaO2/FiO2, ratio of partial pressure of arterial oxygen to fraction of inspired oxygen; ARDS, acute respiratory distress syndrome.
