# Supplementary material for: Efficacy of Thymosin Alpha 1 in the Treatment of COVID-19: A Multicenter Cohort Study
Source: Front Immunol. 2021 Aug 2;12:673693. doi: 10.3389/fimmu.2021.673693 (PMC8366398; doi:10.3389/fimmu.2021.673693)
Supplement: Supplementary file 1 [file DataSheet_1.doc]

**Supplementary tables.**

**TABLE S1 |** The number of patients and thymosin α1 use within five study centers.

| **Variables** | **Union Jiangbei Hospital** | **Wuhan No.9 Hospital** | **Wuhan No.4 Hospital** | **Wuhan Jinyintan Hospital** | **Huangshi Central Hospital** | ***p*** |
| --- | --- | --- | --- | --- | --- | --- |
| Number of patients | 200 | 743 | 79 | 1222 | 38 | < 0.001 |
| Tα1 use, n (%) | 0 (0) | 12 (1.6) | 4 (5.0) | 285 (23.3) | 5 (13.1) | < 0.001 |
| Non-recovery rate, n (%) | 39 (18.5) | 98 (13.2) | 21 (26.5) | 176 (14.4) | 21 (55.3) | < 0.001 |

**TABLE S2 |** Interactions between thymosin α1 use and disease severity indexes on non-recovery rate.

| **Subgroups** | **Adjusted ORs (95%CI)** | ***p*** | ***P*_interaction_** |
| --- | --- | --- | --- |
| **Maximum SOFA ≥2** |  |  | 0.010 |
| Yes (n = 992) | 1.7 (1.2 – 2.6) | 0.008 |  |
| No (n = 1290) | 0.4 (0.1 – 1.2) | 0.114 |  |
| **PaO2/FiO2 <300** |  |  | 0.593 |
| Yes (n = 730) | 1.8 (1.0 – 3.2) | 0.043 |  |
| No (n = 1552) | 1.4 (0.8 – 2.2) | 0.133 |  |
| **ICU admission** |  |  | < 0.001 |
| Yes (n = 329) | 6.8 (2.7 – 17.1) | < 0.001 |  |
| No (n = 1953) | 0.9 (0.6 – 1.5) | 0.942 |  |
| **Mechanical ventilation** |  |  | 0.053 |
| Yes (n = 137) | 2.5 (0.4 – 14.2) | 0.274 |  |
| No (n = 2145) | 1.0 (0.6 – 1.6) | 0.805 |  |
| **ARDS** |  |  | 0.045 |
| Yes (n = 603) | 1.6 (1.0 – 2.6) | 0.045 |  |
| No (n = 1679) | 0.4 (0.1 – 1.4) | 0.179 |  |

^Note^: P_interaction_ represented the p value of the interaction between thymosin α1 use and different disease severity indexes. All models were adjusted for age, diabetes, lymphocyte and platelet count, serum creatinine level, corticosteroids and interferon use.

Abbreviations: ORs, odds ratios; PaO2/FiO2, ratio of partial pressure of arterial oxygen to fraction of inspired oxygen; ICU, intensive care unit; SOFA, sequential organ failure assessment.

**TABLE S3 |** Comparisons of covariates after propensity score matching.

| **Variables** | **All patients** | | |  | **Patients with SOFA ≥2** | | |  |
| --- | --- | --- | --- | --- | --- | --- | --- | --- |
|  | **Non-thymosin α1 group**  **(n = 720)** | **Thymosin α1 group**  **(n = 306)** | ***p*** |  | **Non-thymosin α1 group**  **(n = 302)** | **Thymosin**  **α1 group**  **(n = 142)** | ***p*** | |
| Age (>65), n (%) | 232 (32.2) | 93 (30.3) | 0.564 |  | 118 (39.0) | 53 (37.3) | 0.774 | |
| Hypertension, n (%) | 216 (30.0) | 98 (32.0) | 0.519 |  | 96 (31.7) | 52 (36.6) | 0.314 | |
| Chronic heart diseases, n (%) | 40 (5.5) | 18 (5.8) | 0.836 |  | 31 (10.2) | 11 (7.7) | 0.398 | |
| COPD, n (%) | 21 (2.9) | 9 (2.9) | 0.983 |  | 10 (3.3) | 6 (4.2) | 0.630 | |
| ICU admission, n (%) | 89 (12.37) | 42 (13.7) | 0.549 |  | 66 (21.8) | 36 (25.3) | 0.414 | |
| Maximum SOFA, (median (IQR)) | 1 (0 – 2) | 1 (0 – 3) | 0.406 |  | 3 (2 – 5) | 3 (2 – 5) | 0.071 | |
| Initial white blood cell (10^9/L) | 6.6 ± 3.5 | 6.9 ± 4.0 | 0.294 |  | 7.4 ± 4.5 | 8.0 ± 4.8 | 0.167 | |
| Initial lymphocyte cell (10^9/L) | 1.0 ± 0.6 | 1.0 ± 0.5 | 0.472 |  | 0.8 ± 0.4 | 0.8 ± 0.5 | 0.768 | |
| Initial platelet count (10^9/L) | 213.7 ± 91.3 | 207.5 ± 87.0 | 0.317 |  | 187.1 ± 85.3 | 183.5 ± 79.7 | 0.680 | |
| Initial hemoglobin level (g/L) | 123.8 ± 16.5 | 123.4 ± 18.2 | 0.734 |  | 123.2 ± 17.3 | 121.3 ± 19.5 | 0.309 | |
| Initial serum creatinine (mmol/dL) | 81.2 ± 80.1 | 84.5 ± 78.7 | 0.414 |  | 84.8 ± 88.5 | 100.1 ± 110.5 | 0.119 | |

Abbreviations: SOFA, sequential organ failure assessment; COPD, chronic obstructive pulmonary disease; ICU, intensive care unit; ARDS, acute respiratory distress syndrome; AKI, acute kidney injury.

**TABLE S4 |** Sensitivity analysis using different dependent outcomes in logistic models.

| **Variables** | **Model 1** | | **Model 2** | | **Model 3** | |
| --- | --- | --- | --- | --- | --- | --- |
|  | **OR (95% CI)** | ***p*** | **OR (95% CI)** | ***p*** | **OR (95% CI)** | ***p*** |
| **Pattern 1** |  |  |  |  |  |  |
| Thymosin α1 use | 1.6 (1.2 – 2.1) | 0.001 | 1.4 (1.1 – 2.1) | 0.036 | 1.4 (0.8 – 2.2) | 0.057 |
| **Pattern 2** |  |  |  |  |  |  |
| Thymosin α1 use | 1.5 (1.1 – 2.1) | 0.003 | 1.5 (1.1 – 2.1) | 0.028 | 1.4 (0.9 – 2.3) | 0.118 |
| **Pattern 3** |  |  |  |  |  |  |
| Thymosin α1 use | 1.6 (1.2 – 2.2) | 0.001 | 1.4 (1.1 – 2.0) | 0.034 | 1.4 (0.8 – 2.1) | 0.162 |

Note: In the non-recovery group (n = 355), there are 18 patients still under treatment, but in a severely deteriorated condition during data extraction. For robustness, we performed sensitivity analysis under three patterns, and “death” was used as the dependent outcome. In pattern 1, these 18 patients were divided into the "alive" group. In pattern 2, these 18 patients were divided into the "death" group. In pattern 3, these 18 patients were excluded from the analysis. The results were stable in all these three patterns.

Model 1: crude OR. Model 2: adjusted for age, diabetes mellitus, PaO2/FiO2, lymphocyte, platelet, creatinine level, hospital center. Model 3: confounders in model 2 + maximum SOFA score.

Abbreviations: OR, odds ratio; aORs, adjusted odds ratios; PaO2/FiO2, ratio of partial pressure of arterial oxygen to fraction of inspired oxygen; SOFA, sequential organ failure assessment.

**TABLE S5 |** Interactions between thymosin α1 use and disease severity indexes on in-hospital mortality.

| **Subgroups** | **Crude ORs (95%CI)** | ***p*** | **Adjusted ORs (95%CI)** | ***p*** | ***P*_interaction_** |
| --- | --- | --- | --- | --- | --- |
| **Maximum SOFA ≥2** |  |  |  |  | 0.009 |
| Yes (n = 992) | 2.1 (1.4 – 3.0) | < 0.001 | 1.7 (1.1 – 2.7) | 0.008 |  |
| No (n = 1290) | 0.3 (0.1 – 1.2) | 0.102 | 0.4 (0.1 – 1.7) | 0.280 |  |
| **PaO2/FiO2 <300** |  |  |  |  | 0.124 |
| Yes (n = 730) | 2.0 (1.2 – 3.4) | 0.005 | 1.3 (0.7 – 2.4) | 0.394 |  |
| No (n = 1552) | 1.7 (1.1 – 2.6) | 0.005 | 1.5 (0.9 – 2.4) | 0.087 |  |
| **ICU admission** |  |  |  |  | 0.034 |
| Yes (n = 329) | 5.0 (2.3 – 10.9) | < 0.001 | 3.4 (1.3 – 8.6) | 0.007 |  |
| No (n = 1953) | 1.3 (0.8 – 2.0) | 0.156 | 0.9 (0.5 – 1.5) | 0.877 |  |
| **Mechanical ventilation** |  |  |  |  | 0.461 |
| Yes (n = 137) | 3.1 (0.8 – 11.3) | 0.073 | 0.8 (0.1 – 4.5) | 0.826 |  |
| No (n = 2145) | 1.3 (0.8 – 1.9) | 0.175 | 0.8 (0.6 – 1.6) | 0.847 |  |
| **ARDS** |  |  |  |  | 0.691 |
| Yes (n = 603) | 1.5 (0.9 -2.3) | 0.051 | 0.9 (0.5 – 1.5) | 0.708 |  |
| No (n = 1679) | 0.6 (0.2 – 2.1) | 0.494 | 0.3 (0.1 – 1.3) | 0.143 |  |

Note: hospital mortality was used as the dependent variable. *^P^*_interaction_ represented the p value of the interaction between thymosin α1 use and different disease severity indexes.

Abbreviations: ORs, odds ratios; PaO2/FiO2, ratio of partial pressure of arterial oxygen to fraction of inspired oxygen; ICU, intensive care unit; SOFA, sequential organ failure assessment.

**TABLE S6 |** Association between the duration/timing of thymosin α1 use and non-recover rate (including SOFA).

| **Duration of thymosin α1 use (days)** | **ORs** | **95%CI** | ***p*** | **Timing of thymosin α1 use (days)** | **ORs** | **95%CI** | ***p*** |
| --- | --- | --- | --- | --- | --- | --- | --- |
| **Model 1** | | | | **Model 1** | | | |
| Continuous variable  (n = 306) | 1.0 | 0.9 – 1.1 | 0.312 | Continuous variable (n = 306) | 1.1 | 1.0 – 1.2 | <0.001 |
| **Model 2** (median value) | | | | **Model 2** (median value) | | | |
| ≤5 (n = 172) | Ref. |  |  | ≤3 (n = 159) | Ref. |  |  |
| >5 (n = 134) | 1.2 | 0.5 – 3.0 | 0.634 | >3 (n = 147) | 2.5 | 1.0 – 6.3 | 0.050 |
| **Model 3** (tertile analysis) (*p* for trend: 0.504) | | | | **Model 3** (tertile analysis) (*p* for trend: 0.005) | | | |
| ≤3 (n = 106) | Ref. |  |  | ≤1 (n = 109) | Ref. |  |  |
| 3 -7 (n = 103) | 0.1 | 0.03 – 0.6 | 0.007 | 2 -10 (n = 91) | 7.9 | 1.7 – 36.5 | 0.008 |
| ≥7 (n = 97) | 1.1 | 0.4 – 3.3 | 0.800 | ≥10 (n = 106) | 8.3 | 1.9 – 35.3 | 0.004 |

Note: all models were adjusted for age, diabetes, PaO2/FiO2, lymphocyte and platelet count, serum creatinine level and maximum SOFA score.

Abbreviations: ORs, odds ratios.

**Supplementary Figure Legends**

**
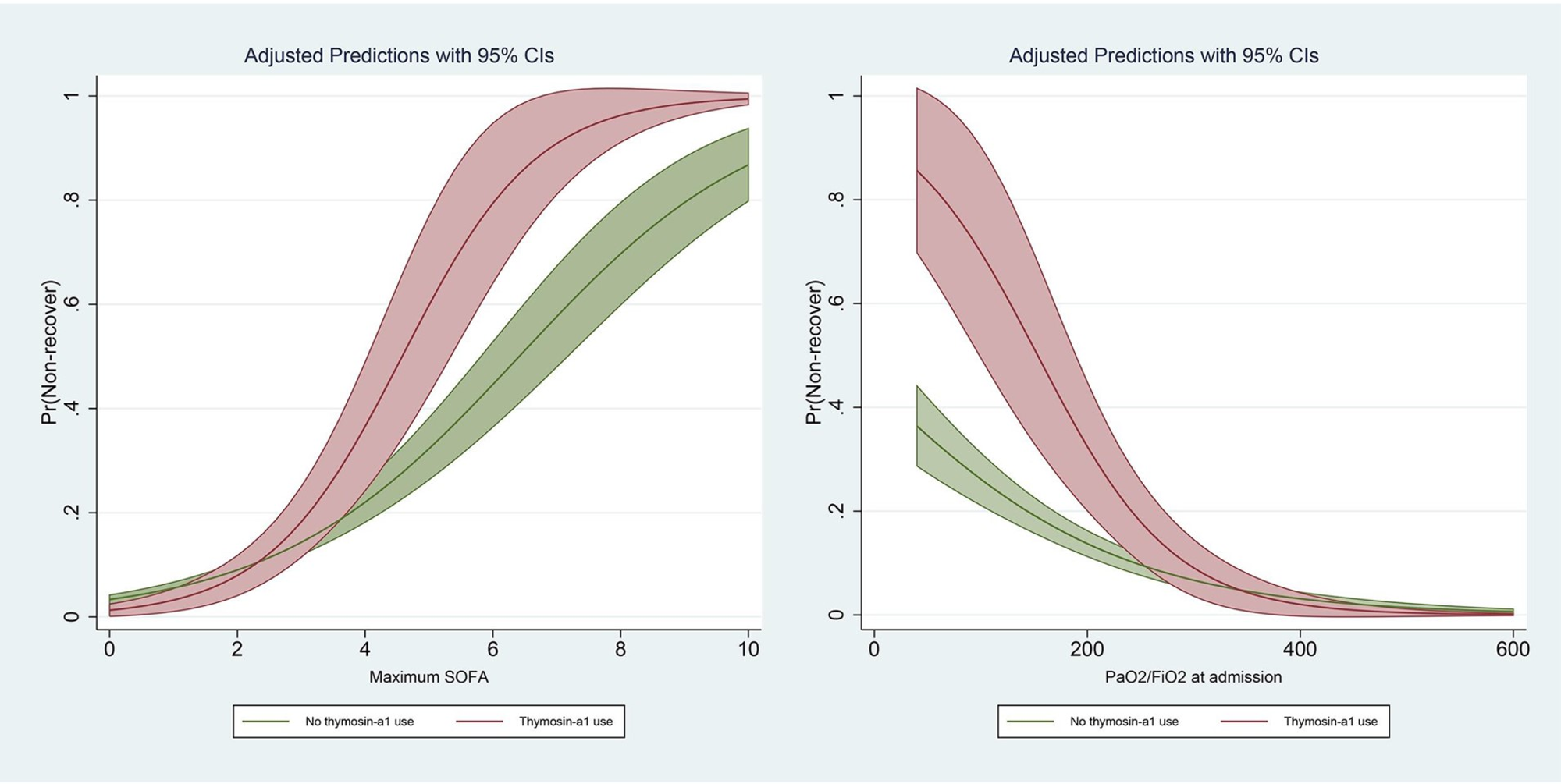
**

**FIGURE S1**. Predicted marginal effect of thymosin-a1 therapy at different SOFA scores and PaO2/FiO2 values.

Abbreviations: SOFA, sequential organ failure assessment; PaO2/FiO2, ratio of partial pressure of arterial oxygen to fraction of inspired oxygen.


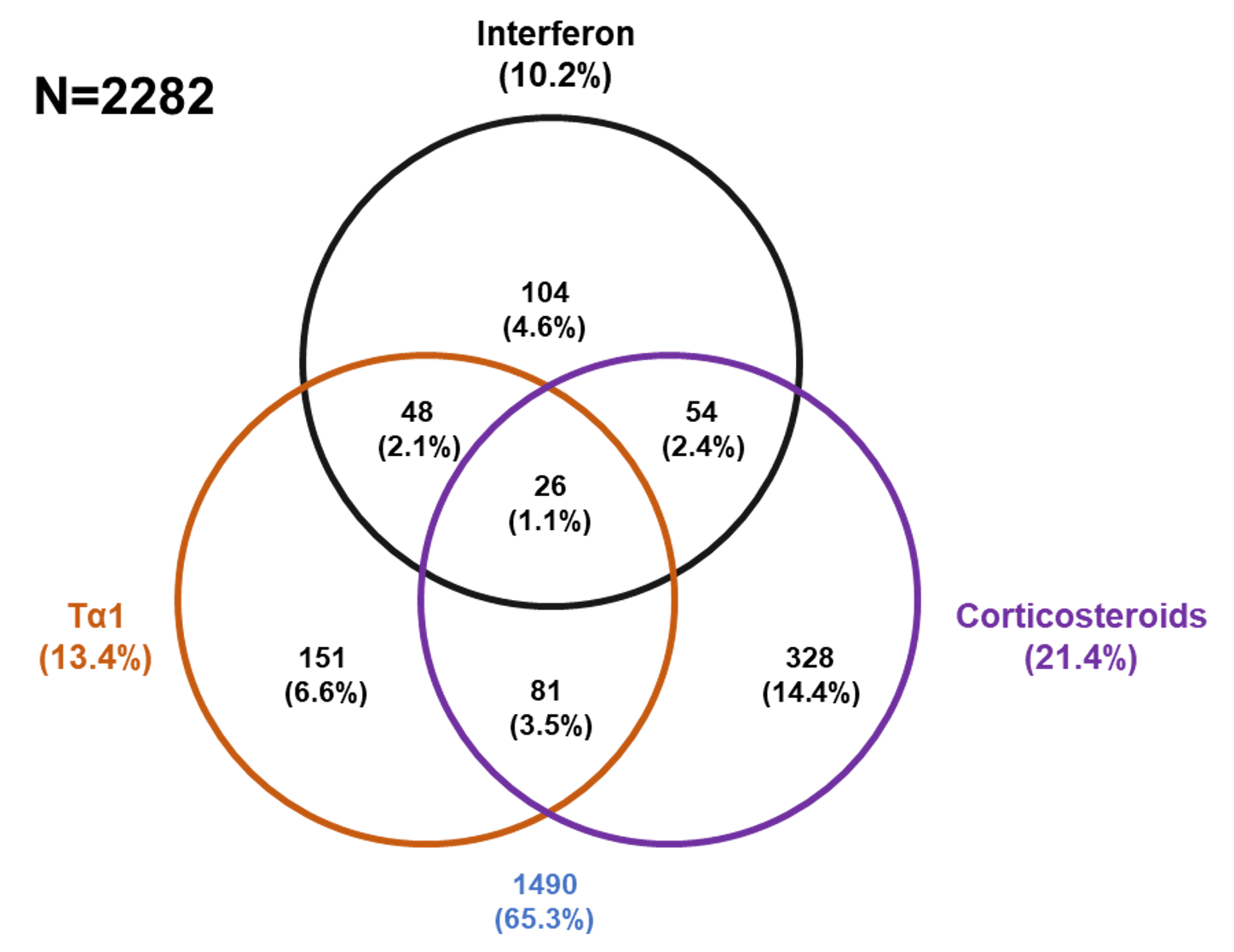


**FIGURE S2**. Venn diagram of the number of patients who received Tα1, interferon and corticosteroid therapies.

Note: The numbers within each region represent the number of patients who received that therapy combination. For example, 81(3.5%) patients received Tα1 and corticosteroid therapies.
